# Intragastric Nonabsorbable Sutures From Prior Endoscopic Sleeve Gastroplasty Discovered During Robotic Hiatal Hernia Repair: A Case Report

**DOI:** 10.7759/cureus.107197

**Published:** 2026-04-16

**Authors:** Peter Hsin, Samantha Siu, Bieu Mach, Ashwin Mahendra, Jorge Rabaza

**Affiliations:** 1 Medicine, Florida International University, Herbert Wertheim College of Medicine, Miami, USA; 2 General Surgery, Baptist Health South Florida, Miami, USA

**Keywords:** endoscopic sleeve gastroplasty, endoscopic suturing, gastric plication, hiatal hernia repair, intragastric foreign body

## Abstract

Endoscopic sleeve gastroplasty (ESG) has emerged as a minimally invasive bariatric procedure that utilizes endoscopic suturing to reduce gastric volume. While generally considered safe, delayed complications related to retained suture material are incompletely characterized in the literature. We report a case of intragastric nonabsorbable sutures discovered during revisional hiatal hernia surgery in a patient with a remote history of prior foregut intervention.

A 58-year-old woman with a history of hiatal hernia repair with fundoplication approximately 10 years prior presented for surgical evaluation of recurrent gastrointestinal symptoms, including postprandial abdominal discomfort, bloating, and prior episodes of melena. Upper endoscopy demonstrated abnormal gastric anatomy, gastritis, and a nodular lesion associated with exposed suture material within the gastric body. Imaging and endoscopic findings raised concern for recurrent hiatal hernia with possible failed fundoplication. The patient subsequently underwent laparoscopic robotic hiatal hernia repair with intraoperative endoscopy.

During intraoperative endoscopic evaluation, multiple intraluminal nonabsorbable sutures were identified extending from the gastric antrum to the fundus. These sutures were protruding into the gastric lumen and appeared consistent with retained material from a prior endoscopic gastric plication procedure, most likely ESG. The sutures were excised and removed prior to completion of the hiatal hernia repair. Pathologic examination confirmed intragastric foreign bodies composed of suture material. The patient tolerated the procedure well and was discharged on postoperative day one without complications.

This case highlights a rare delayed presentation of intragastric suture foreign bodies likely originating from prior endoscopic gastric plication. Awareness of this potential long-term complication is important for surgeons and gastroenterologists managing patients with prior endoscopic bariatric interventions.

## Introduction

Hiatal hernia repair with fundoplication remains a well-established surgical treatment for symptomatic gastroesophageal reflux disease and paraesophageal hernias. Despite generally favorable outcomes, recurrent symptoms may occur due to factors including slipped fundoplication, recurrent hiatal hernia, or anatomical distortion of the gastroesophageal junction [1].

In recent years, endoscopic bariatric interventions have gained increasing popularity as minimally invasive alternatives to surgical weight-loss procedures. Endoscopic sleeve gastroplasty (ESG) utilizes endoscopic suturing systems to create a restrictive gastric sleeve through full-thickness plication of the gastric wall [1-4]. Randomized and multicenter studies have demonstrated its safety and efficacy in achieving weight loss with relatively low complication rates [2-4].

Reported complications of ESG include bleeding, perigastric fluid collections, and gastric perforation, with most events occurring in the early post-procedural period [4,5]. However, long-term data regarding delayed complications, particularly those related to retained suture material, remain limited. In some cases, nonabsorbable sutures may protrude into the gastric lumen and present as mucosal abnormalities or intragastric foreign bodies on endoscopy.

We present a case of delayed intragastric nonabsorbable sutures, likely originating from a prior endoscopic gastric plication procedure, that were incidentally identified during robotic laparoscopic repair of a recurrent hiatal hernia. This case highlights a rare delayed complication of endoscopic gastric plication and underscores the importance of careful intraoperative evaluation in patients with complex foregut surgical histories. Retained or exposed suture material may act as a chronic intragastric foreign body, potentially leading to mucosal irritation, ulceration, bleeding, or nonspecific gastrointestinal symptoms, underscoring the clinical relevance of this finding.

## Case presentation

A 58-year-old female with a history of hiatal hernia repair with fundoplication approximately 10 years prior presented for surgical follow-up with recurrent upper gastrointestinal symptoms. She reported intermittent postprandial right upper quadrant discomfort and abdominal bloating. She also described three prior episodes of melena occurring intermittently over the past several years following her prior foregut surgery, including one episode requiring hospitalization, after which she was discharged on pantoprazole therapy. The exact timing of any prior endoscopic intervention was unclear due to limited available records, though findings suggested a remote procedure.

**Figure 1:**
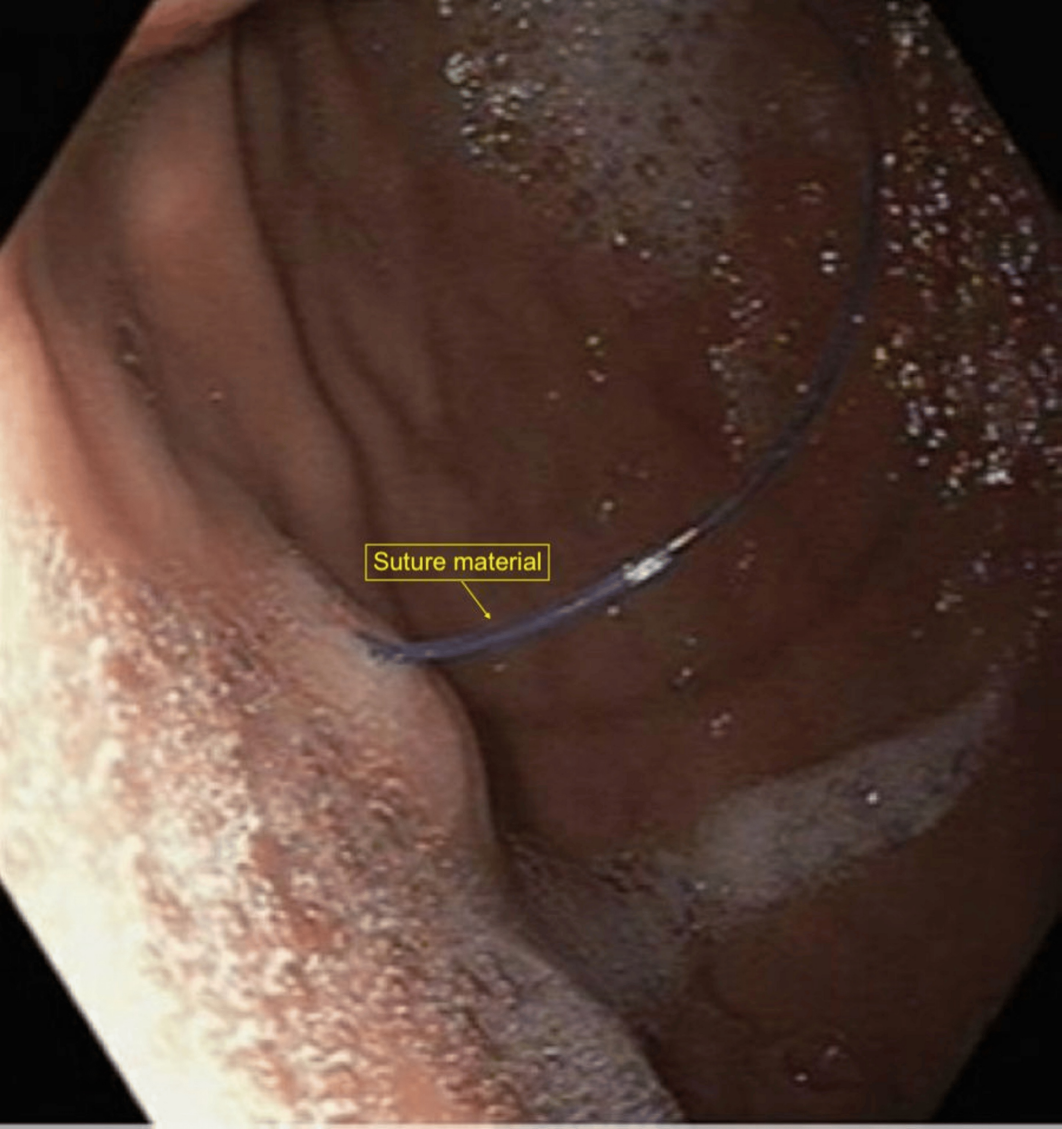
Suture in gastric body Endoscopic image demonstrating a blue nylon suture within the gastric body.

Given her persistent symptoms, she underwent upper endoscopy. Endoscopic evaluation demonstrated mucosal inflammation within the gastric body and antrum characterized by congestion and erythema. Two small gastric polyps measuring approximately 2 mm were identified in the gastric body and biopsied. A solitary papular lesion was also noted in the gastric body and appeared to be associated with exposed nonabsorbable suture material (Figure 1).

**Figure 2:**
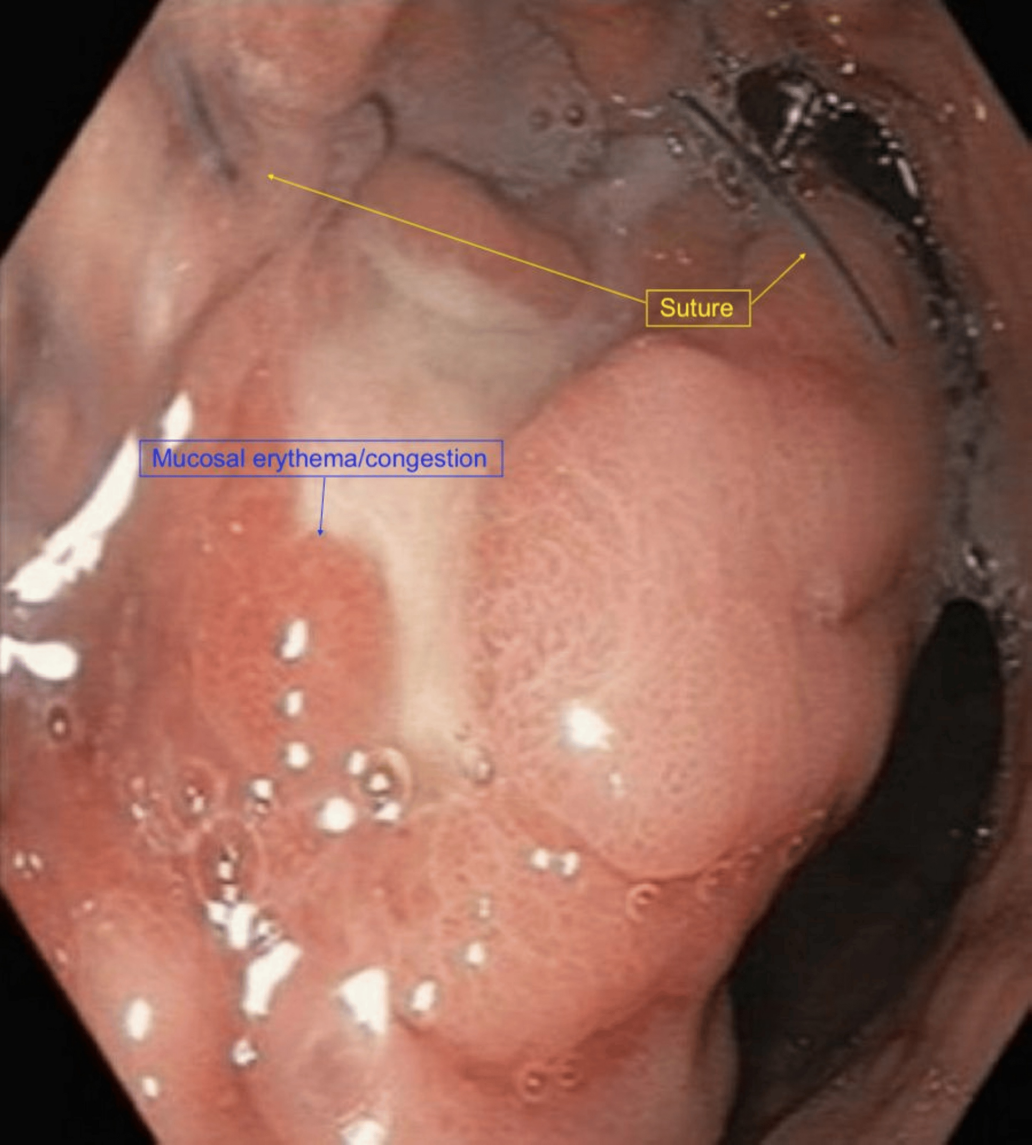
Gastric ulcer with retained suture material Esophagogastroduodenoscopy (EGD) demonstrating a gastric ulcer with surrounding mucosal erythema and congestion. Blue nylon suture material is visible within the affected area.

**Figure 3:**
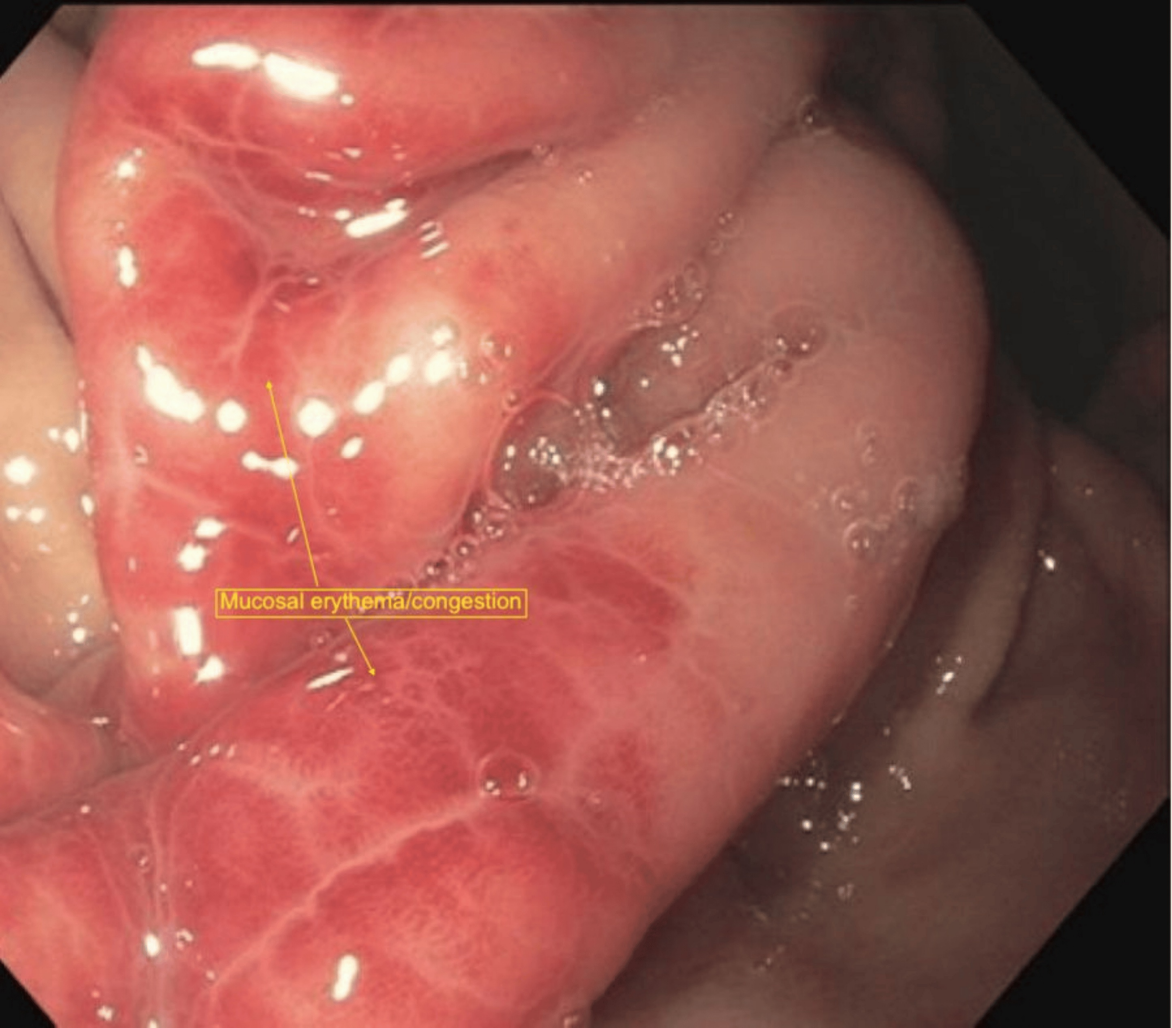
Gastric ulcer Mucosal erythema and congestion consistent with inflammatory process due to retained foreign material leading to a gastric ulcer visualized on Esophagogastroduodenoscopy (EGD).

**Figure 4:**
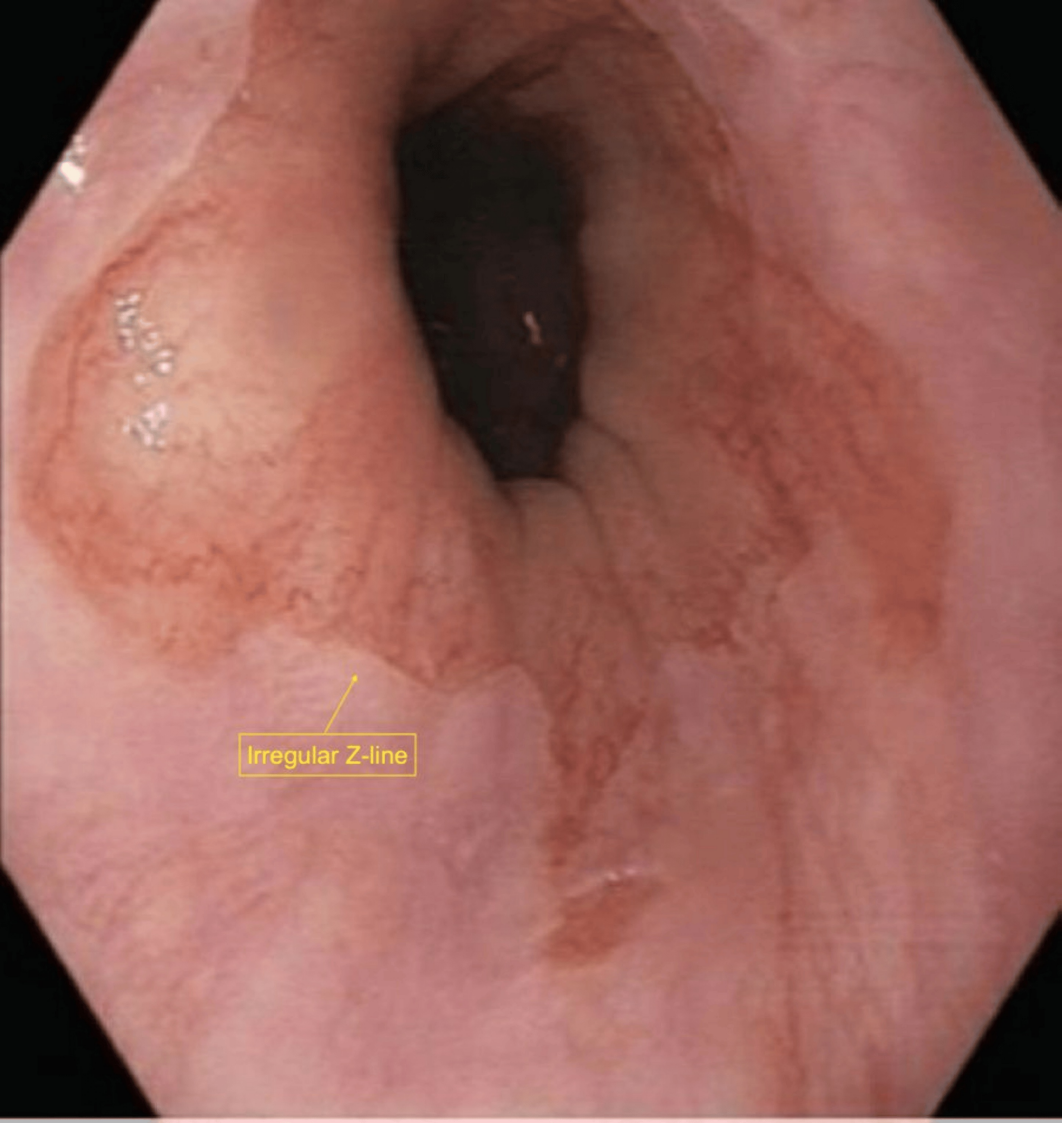
Irregular Z-line Esophagogastroduodenoscopy (EGD) demonstrating an irregular Z-line (squamocolumnar junction) consistent with chronic inflammatory changes.

**Figure 5:**
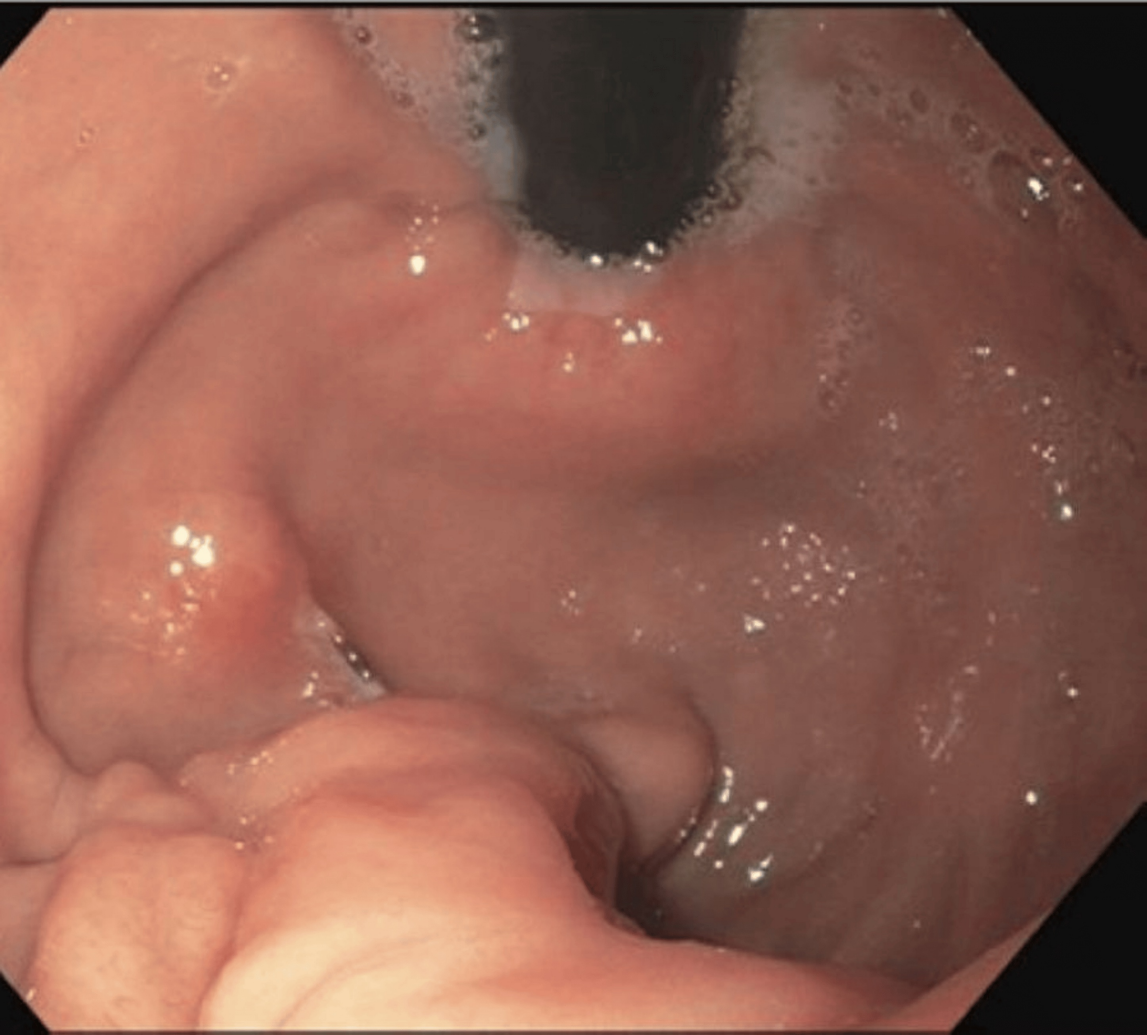
Hiatal hernia Esophagogastroduodenoscopy (EGD) demonstrating proximal displacement of gastroesophageal junction consistent with hiatal hernia.

Additional findings included localized erythema and a thickened fold within the gastric fundus, as well as mild erythematous mucosa in the distal esophagus. A gastric ulcer with surrounding mucosal erythema and congestion was also visualized, with retained suture material present within the lesion (Figure 2). Additional areas of mucosal erythema and congestion consistent with inflammatory changes were noted (Figure 3). Examination of the gastroesophageal junction demonstrated an irregular Z-line suggestive of chronic inflammatory changes (Figure 4), along with proximal displacement of the gastroesophageal junction consistent with a hiatal hernia (Figure 5).

These findings were interpreted as consistent with distorted gastric anatomy, likely related to a recurrent hiatal hernia and prior surgical or endoscopic intervention. Based on these findings, operative management was planned.

Physical examination

On presentation, the patient was afebrile and hemodynamically stable. Vital signs included temperature 36.1°C, heart rate 78 beats per minute, blood pressure 117/81 mmHg, and oxygen saturation of 97% on room air. Her body mass index was 26.45 kg/m².

Physical examination revealed a well-appearing patient in no acute distress. Cardiopulmonary examination was unremarkable. Abdominal examination demonstrated a soft, non-distended abdomen without tenderness, guarding, or rebound. No masses or organomegaly were appreciated.

Operative findings

The patient underwent laparoscopic robotic hiatal hernia repair with intraoperative endoscopy under general anesthesia. After establishing pneumoperitoneum and port placement, adhesiolysis was performed to mobilize the gastroesophageal junction. The hiatal hernia sac was reduced, and circumferential esophageal dissection was completed.

Intraoperative endoscopy revealed multiple intraluminal nonabsorbable sutures extending from the gastric antrum to the fundus. These sutures were protruding into the gastric lumen and appeared consistent with retained suturing material from a prior endoscopic gastric plication procedure. The configuration and distribution of sutures were felt intraoperatively by the surgical team to be most consistent with a prior endoscopic suturing intervention, such as ESG, although no prior procedural records were available for confirmation.

Additional sutures described intraoperatively as V-Loc and STRATAFIX were identified in separate regions, both of which are synthetic barbed sutures commonly used in minimally invasive procedures. In contrast, the sutures visualized endoscopically within the gastric lumen, as shown in the figures, were consistent with blue nylon suture material.

Given these findings, the sutures were carefully excised and removed endoscopically. Removal of foreign material is recommended when feasible due to the risk of mucosal injury and associated complications [6]. The hiatal defect was then repaired using nonabsorbable sutures. Estimated blood loss was minimal, and no intraoperative complications occurred.

Pathologic findings

Gross examination demonstrated multiple fragments of suture material without associated tissue. Findings were consistent with intragastric foreign bodies composed of nonabsorbable sutures.

Postoperative course

The patient tolerated the procedure well and was discharged on postoperative day one without complications. At follow-up, she reported resolution of symptoms and was tolerating a puréed diet. A mild contact dermatitis at trocar sites was treated conservatively.

## Discussion

ESG is an increasingly utilized endoscopic bariatric technique supported by randomized trials and large multicenter studies demonstrating its safety and effectiveness [2-4]. The procedure relies on nonabsorbable sutures to maintain long-term gastric remodeling, which may predispose patients to delayed complications related to retained material.

While early complications such as bleeding and perforation are well described [4,5], delayed complications remain less clearly characterized. Retained or exposed sutures may act as intragastric foreign bodies, leading to mucosal irritation, ulceration, or gastrointestinal bleeding. Management of such foreign bodies typically involves endoscopic removal when feasible, in accordance with established guidelines [6]. Endoscopic suturing systems, such as the OverStitch device, commonly utilize durable suture materials designed to maintain gastric plication, which may contribute to delayed intraluminal exposure or foreign body formation.

Foreign bodies within the gastrointestinal tract can present with a wide range of symptoms, including pain, bleeding, or obstruction, depending on their location and chronicity [7]. In this case, the presence of intragastric sutures likely contributed to the patient’s prior episodes of melena and gastritis.

Retained suture material can lead to mucosal injury through repeated mechanical irritation, disruption of the epithelial barrier, and localized inflammatory response. Over time, this may result in chronic gastritis, mucosal erosion, and ulcer formation, which may clinically manifest as abdominal discomfort or gastrointestinal bleeding, as seen in this case.

This case also highlights the complexity of evaluating recurrent symptoms following prior foregut surgery. Failure of antireflux procedures may result from anatomical disruption or previously unrecognized interventions, complicating both diagnosis and management [8].

The incidental discovery of retained intragastric sutures during revisional hiatal hernia repair underscores the importance of maintaining a broad differential diagnosis in patients with prior endoscopic or surgical interventions. Intraoperative endoscopy played a critical role in both diagnosis and definitive management in this case. The differential diagnosis for this patient’s symptoms and endoscopic findings included recurrent hiatal hernia with failed fundoplication, peptic ulcer disease, gastritis related to medication use, and mucosal injury from prior surgical intervention. The identification of retained intragastric suture material provided a unifying explanation for the observed mucosal inflammation and ulceration, likely due to chronic mechanical irritation and foreign body reaction. In this case, the distribution and configuration of sutures, along with the absence of alternative sources, made prior endoscopic suturing the most likely etiology.

A limitation of this case is the absence of prior procedural documentation confirming a history of ESG or gastric plication. The presumed etiology is based on intraoperative findings and the characteristic distribution and configuration of sutures, which were deemed by the surgical team to be consistent with endoscopic suturing techniques. However, alternative sources of retained suture material cannot be definitively excluded.

To our knowledge, reports of delayed intragastric nonabsorbable sutures identified during unrelated revisional foregut surgery remain exceedingly rare, further emphasizing the clinical relevance of this case.

## Conclusions

Delayed intragastric foreign bodies composed of nonabsorbable sutures may occur following endoscopic gastric plication procedures, including ESG, although definitive attribution may not always be possible in the absence of prior procedural documentation. These sutures may protrude into the gastric lumen and present with symptoms such as gastritis or gastrointestinal bleeding.

In patients with a history of endoscopic bariatric interventions who present with recurrent upper gastrointestinal symptoms, retained suture material should be considered as a potential etiology. Intraoperative endoscopy plays a critical role in the identification and management of these complications.

Recognition of this rare delayed complication may improve diagnostic accuracy and guide appropriate management in patients with complex foregut surgical histories.

## References

[1] de Moura DT, de Moura EG, Thompson CC (2019). Endoscopic sleeve gastroplasty: from whence we came and where we are going. World J Gastrointest Endosc.

[2] Abu Dayyeh BK, Bazerbachi F, Vargas EJ (2022). Endoscopic sleeve gastroplasty for treatment of class 1 and 2 obesity (MERIT): a prospective, multicentre, randomised trial. Lancet.

[3] Barrichello S, Hourneaux de Moura DT, Hourneaux de Moura EG (2019). Endoscopic sleeve gastroplasty in the management of overweight and obesity: an international multicenter study. Gastrointest Endosc.

[4] Alqahtani A, Al-Darwish A, Mahmoud AE, Alqahtani YA, Elahmedi M (2019). Short-term outcomes of endoscopic sleeve gastroplasty in 1000 consecutive patients. Gastrointest Endosc.

[5] Surve A, Cottam D, Medlin W, Richards C, Belnap L (2019). A video case report of gastric perforation following endoscopic sleeve gastroplasty and its surgical treatment. Obes Surg.

[6] Birk M, Bauerfeind P, Deprez PH (2016). Removal of foreign bodies in the upper gastrointestinal tract in adults: European Society of Gastrointestinal Endoscopy (ESGE) clinical guideline. Endoscopy.

[7] Egberts K, Brown WA, O'Brien PE (2011). Systematic review of erosion after laparoscopic adjustable gastric banding. Obes Surg.

[8] Patti MG, Allaix ME, Fisichella PM (2015). Analysis of the causes of failed antireflux surgery and the principles of treatment: a review. JAMA Surg.

